# Intratumoral microbiota: implications for cancer onset, progression, and therapy

**DOI:** 10.3389/fimmu.2023.1301506

**Published:** 2024-01-16

**Authors:** Jinmei Wu, Pengfei Zhang, Wuxuan Mei, Changchun Zeng

**Affiliations:** ^1^ Department of Medical Laboratory, Shenzhen Longhua District Central Hospital, Shenzhen, China; ^2^ Xianning Medical College, Hubei University of Science and Technology, Xianning, China

**Keywords:** intratumoral microbiota, immunotherapy, cancer, treatment, tumor microenvironment

## Abstract

Significant advancements have been made in comprehending the interactions between the microbiome and cancer. However, prevailing research predominantly directs its focus toward the gut microbiome, affording limited consideration to the interactions of intratumoral microbiota and tumors. Within the tumor microenvironment (TME), the intratumoral microbiome and its associated products wield regulatory influence, directing the modulation of cancer cell properties and impacting immune system functionality. However, to grasp a more profound insight into the intratumoral microbiota in cancer, further research into its underlying mechanisms is necessary. In this review, we delve into the intricate associations between intratumoral microbiota and cancer, with a specific focus on elucidating the significant contribution of intratumoral microbiota to the onset and advancement of cancer. Notably, we provide a detailed exploration of therapeutic advances facilitated by intratumoral microbiota, offering insights into recent developments in this burgeoning field.

## 1 Introduction

The presence of numerous microorganisms such as viruses, bacteria, fungi, and other microbes within the human body is vital for human health. These microorganisms exhibit colonization patterns in multiple anatomical sites, encompassing the oral cavity, skin, gastrointestinal tract, respiratory tract, and genitalia. Symbiotic interactions between humans and their microbiome are critical and contribute significantly to human health (1–3). Extensive inquiries into the human microbiome have illuminated variations in the microbial communities among individuals in a state of health and those experiencing pathological conditions. Moreover, the microbiome is closely linked to cancer by influencing the carcinogenesis process in the human body (4). The well-documented link between cancer and specific viruses, such as Epstein-Barr virus and human papillomavirus, underscores their potential to initiate oncogenic activation (5). Oncoviral infections have been shown to promote tumorigenesis by enabling the incorporation of oncogenes within the human genome structure (6, 7).

Research into host-microbial interactions has notably propelled the comprehension of intratumoral microbiota (8, 9). The advancement of detection technologies and enhanced comprehension of the TME have substantiated the presence of intratumoral bacteria. Tumor tissue presents a significantly reduced presence of microbial and fungal biomass when compared to the abundance observed in the gut environment (10, 11). Recent findings point to exclusive bacterial and fungal patterns characteristic of individual tumor types (12, 13). In comparison to normal tissues, tumor tissues manifested a heightened abundance of bacterial and fungal burdens. Remarkably, a substantial enrichment of multiple bacterial strains was observed specifically within tumor tissues. Intratumoral microbial components, distinguished in several tumor types, manifest meaningful correlations with the onset and advancement of cancer (14, 15). Recent studies underscore the fundamental importance of gut microbiota in governing the immune responses. Additionally, it has been demonstrated that the microbiota present within tumors can significantly shape the local immune responses in the TME, potentially affecting tumor progression (16). Within the TME, intratumoral microbiota conspicuously demonstrate anti-tumorigenic manifestations by orchestrating heightened antigen presentation, activating T and NK cells, executing proficient immunosurveillance, and synthesizing metabolites that suppress tumor progression. Conversely, pro-tumorigenic effects are characterized by elevated levels of reactive oxygen species (ROS), the emergence of driver mutations, the inactivation of T cells, and the induction of immunosuppression (3). The intratumoral microbiota manifests varied roles in anti-tumor immunity, with the potential to either enhance or suppress anti-tumor immune responses (17). Consequently, these roles have implications for the effectiveness of immunotherapy (16, 18). In recent years, there has been a surge in research interest delving into the intricate interplay between gut microbiota and the etiology as well as therapeutic responses in cancer. Nonetheless, increasing attention is being paid to intratumoral microbiota (3).

This review presents a thorough analysis of the burgeoning field of intratumoral microbiota research. We delve into its origins, the rich spectrum of its diversity, the intriguing links between intratumoral and gut microbiota, mechanistic involvement in tumorigenesis, and the exciting potential it holds for innovative tumor therapeutics. This review offers promising avenues for developing innovative therapeutic interventions leveraging intratumoral microbiota toward effective tumor management.

## 2 Intratumoral microbiota: unveiling their features

### 2.1 Origin of intratumoral microbiota

**Figure 1:**
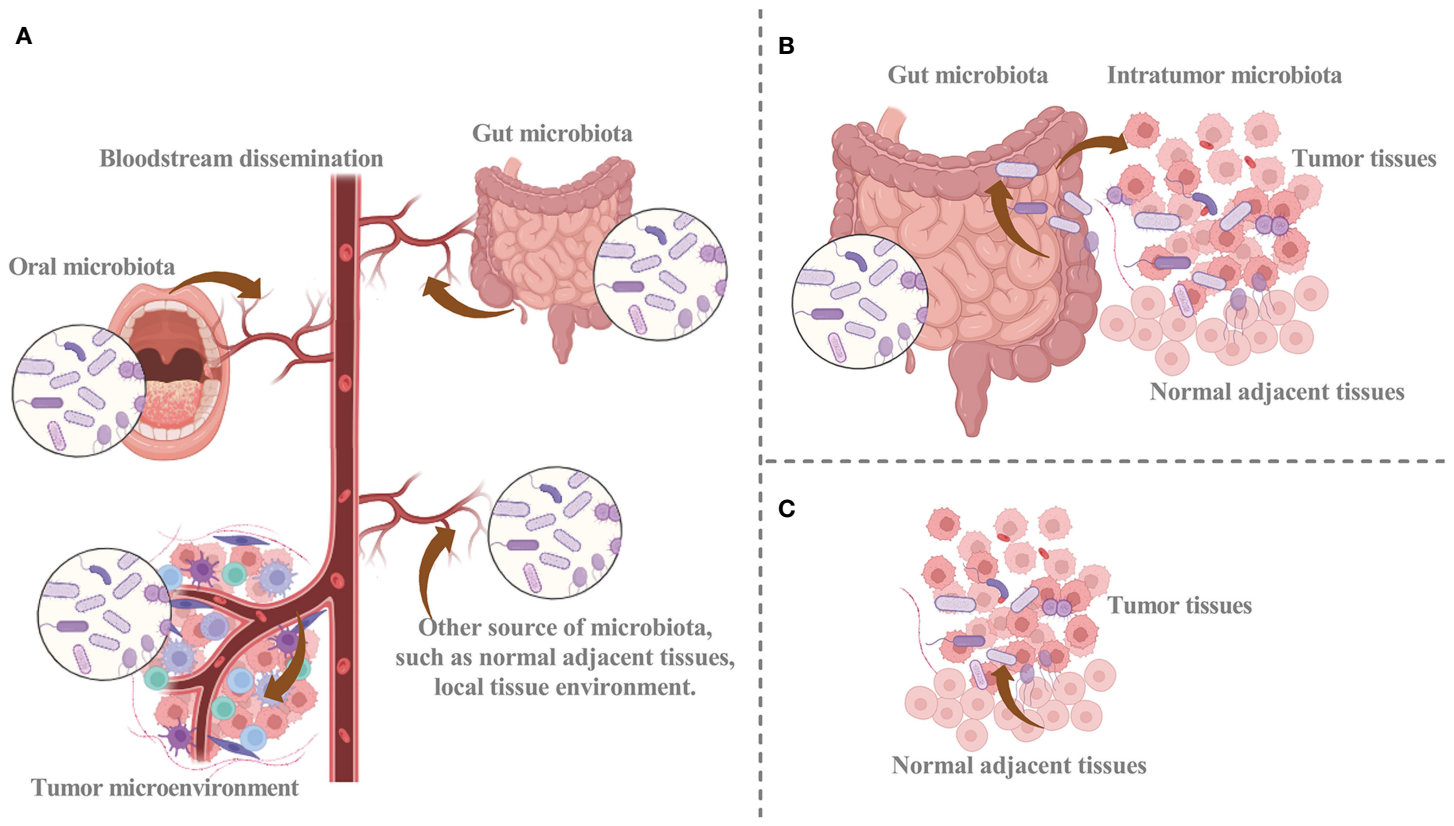
The potential sources of intratumoral microbiota. **(A)** Hematogenous spread facilitates the infiltration of intratumor microbes from oral, intestinal, and other sources into tumor sites. **(B)** Microbiota can disrupt the mucosal barrier and infiltrate tumor sites, and intratumoral microbiota of cancer may infiltrate tumor sites via the duct. **(C)** Normal adjacent tissue may provide a source for intratumor microbiota. Graphics created with BioRender.com.

Despite the significant attention given to intratumoral microbiota, their origins have not been fully elucidated. Recent research has revealed that intratumoral microbiota may arise from distinct sources (**Figure 1**) (3, 11, 19, 20). The intratumoral microbiota may arise from breaches in mucosal barriers. Intratumoral microbiota is commonly found in cancers originating at mucosal sites, including colorectal, pancreatic, cervical, and lung cancer (21). These organs have externally exposed cavities, and the mucosal destruction that occurs during tumorigenesis can provide a pathway for microorganisms colonizing the mucosa to invade the tumor. Thus, the breach of mucosal barriers, with other factors, may lead to the colonization of microbiota in the TME and facilitate their complex interactions (16, 22). The identified representative bacteria within nasopharyngeal carcinoma tissues exhibit approximately 69% similarity in single-nucleotide variations to bacteria present in the nasopharyngeal microbiota. Subsequently, resemblances are observed with bacteria from the oral cavity (24.1%) and the gut (6.9%). These findings unequivocally establish the nasopharyngeal microbiota as the primary reservoir of intratumoral bacteria within nasopharyngeal carcinoma (23). Although there are abundant microbiomes in human mucosal organs, the idea that intratumoral microbiota can only come from the mucosal site through the mucosal barrier cannot explain all the intratumoral microbiota. A portion of intratumoral bacteria is rare within the mucosal organs of the corresponding tumors, while others are prevalent in non-mucosal origin tumors, such as breast cancer, suggesting other potential sources of intratumoral microbiota (11, 24). Therefore, additional investigation is necessary to clarify the mechanisms that facilitate microbial infiltration from mucosal organs into the TME.

The circulatory system represents another potential origin for intratumoral microbiota (3, 11). The chemotactic gradient of necrotic cell debris within a tumor is a mechanism that attracts microorganisms from different locations into the blood circulation. Malformed blood vessels provide a conducive setting for intratumoral microbiota to colonize the TME through hematogenous spread (9). Hematogenous spread facilitates the recruitment of microorganisms from various sites, including the oral cavity and intestines, to the tumor site, where they can colonize the tumor via infiltration through impaired blood vessels. The circulatory system, including blood, lymphatic fluid, and the internal passages of the alimentary tract, provides a plausible pathway for the transfer of microbiota. Considering the anatomical interconnectedness of the oral cavity, respiratory tract, and gastrointestinal tract, it is plausible that oral microbiota can easily migrate to these respective anatomical regions. When the oral microbiota undergoes ecological disruption, they may gain entry into the tumor and convert it into intratumoral microbiota (25).

Bacteria from adjacent normal tissues have been found in organs previously believed to lack microbial presence. Moreover, the bacterial composition within tumor tissues closely resembles that of adjacent normal tissues (3, 11, 26). The significant similarity of microbiota composition between tumor microbiota and normal adjacent tissue microbiota can be explained by the origin of normal adjacent tissue microbiota from TME. Within normal adjacent tissues, microorganisms from blood vessels or mucosal organs may infiltrate the TME stimulated by oxygen and chemotactic gradients (11). In addition, microorganisms in normal tissues may originate from the tumor site. Consequently, it is unclear whether normal adjacent tissues serve as a source of intratumoral microbiota, and further substantiation is necessary to elucidate this matter.

As knowledge of the origin and mechanisms of intratumoral microbiota grows, a more comprehensive understanding of intratumoral microbiota may assist in devising more potent therapeutic approaches. Exploring the various sources of intratumoral microbiota, analyzing their composition, and comparing them with the microbiome of other body sites may facilitate the identification of intratumoral microbiota. Furthermore, investigating the molecular mechanisms that underlie the infiltration of microorganisms into the TME is a compelling area of research.

### 2.2 Diversity of intratumoral microbiota

Given the possibility of multiple origins of intratumoral microbiota, it is plausible to suggest that the microbiome compositions of various cancer types are heterogeneous (15, 27). Within a variety of prevalent cancer types, there are distinct microbial signatures present in tissue and blood samples, each linked to a specific microbiota. Such microbial signatures have been utilized to differentiate healthy individuals from those with cancer, indicating that these signatures may have diagnostic potential (28). The utilization of a rigorous decontamination pipeline in analyzing The Cancer Genome Atlas (TCGA) database at the whole-genome and whole-transcriptome level has allowed for the discovery of unique microbial signatures present in both blood and tumor tissue that was specific to certain cancer types (15, 27). A recent pan-cancer study investigated the presence of cancer-associated fungi in 17,401 samples from 35 distinct cancer types. The findings indicate that fungal DNA and cells exhibit low abundance in several prevalent human cancers, with diverse community compositions across various cancer types. Distinct fungal species and corresponding cellular compositions were associated with specific types of cancer (15). Tumor microbial communities exhibit a predominance of bacteria, with a lower abundance of fungi. The composition of microbial communities in adjacent normal tissues is similar to that of tumor microbial communities. Some microorganisms have been identified in multiple types of tumors, although their abundance can differ depending on the specific cancer type (26).

Intratumoral bacteria possess some common characteristics. Their prevalence within cancerous tissues is significantly lower when compared to that of the gut, with qPCR and imaging quantification indicating that the bacterial presence is discernible in a fraction of cancer cells, varying from 0.1% to 10%. The microbial diversity is generally diminished in cancerous tissue as opposed to normal tissue, suggesting that tumors may foster a distinct milieu that selects for specific bacterial species. The majority of these bacteria are commensal organisms primarily inhabiting the intracellular compartment. The diverse bacterial ecosystems within cancer tissues could potentially contribute to multifunctional mechanisms when interacting with cancerous cells (14, 29).

The microbiota of colorectal cancer has been investigated, with some bacteria like *Bacteroides fragilis*, *Escherichia coli*, and *Fusobacterium nucleatum* frequently detected within tumor tissues. In addition, fungal species, such as Candida albicans, have been detected in some colorectal cancer samples (30–32). *Helicobacter pylori*, a bacterium responsible for chronic gastritis and peptic ulcers, is linked to the heightened risk of developing gastric cancer. Furthermore, some bacterial species like *Streptococcus anginosus* and *Lactobacillus* have been identified in some gastric cancer samples (33, 34). A pan-cancer analysis of the mycobiome across various anatomical locations revealed the presence of tumor-associated *fungi* and a significant abundance of *Candida* in gastrointestinal malignancies. Mycobiome communities in gastrointestinal tumors exhibit a high prevalence of *Cyberlindnera jadinii*, *Saccharomyces cerevisiae*, and *Candida* species. *Blastomyces* species are prevalent within pulmonary carcinomas, while *Malassezia* species are abundant within mammary tumors (13). *Fusobacterium nucleatum*, associated with colorectal tumors, also exhibited a higher prevalence in pancreatic and breast malignancies. Microbial compositions vary distinctly across different subtypes of tumors. For instance, multiple bacterial taxa exhibited distinct prevalence when comparing various subtypes of breast cancer, characterized by their human epidermal growth factor receptor 2 (HER2), estrogen receptor (ER), and progesterone receptor (PR) status. *Granulicatella_Unknown species31* (species) and *Dyadobacter* (genus) exhibit enrichment in HER2+ breast cancer patients. *Corynebacterium* (genus) demonstrates enrichment in ER- breast cancer patients, while *Actinomycetaceae* (family), *Sphingomonas_Unknown species124* (species), *Streptophyta_Unknown genus116* (genus), *Lautropia_Unknown species38* (species), and *Actinomyces odontolyticus* (species) manifest enrichment in ER+ breast cancer patients. *Actinobacteria* (class) displays enrichment in non-triple negative breast cancer. Conversely, *Achromobacter denitrificans* (species), *Bacillus_Unknown species21* (species), *Leptotrichia_Unknown species21* (species), *Streptophyta_Unknown genus116* (genus), *Nocardiopsaceae* (family), and *Achromobacter* (genus) are enriched in triple-negative breast cancer. Moreover, breast tumors exhibited a heightened bacterial abundance in comparison to normal adjacent tissue (14).

### 2.3 The association between intratumoral and gut microbiota

The current research landscape is witnessing a surge in studies exploring the correlation between intratumoral and gut microbiota. Specific bacterial species within the gut microbiota have the potential to infiltrate the intestinal mucosa, enter the bloodstream, and inhabit neoplastic lesions, thus shaping the composition of the microbiota within tumors. The gut microbiota-tumor interplay has emerged as a critical factor influencing the onset and advancement of diverse forms of cancer. In glioma, intratumoral bacteria can originate not only from the gut microbiota but also from the oral cavity or adjacent brain tissue. Glioma-induced shifts in the local microenvironment, involving the disruption of the blood-brain barrier and immunosuppression, create conducive conditions for bacterial infiltration via either hematogenous or neuronal retrograde pathways. It is plausible that these bacteria existed in the brain tissue before tumorigenesis, with those adapting to the TME demonstrating growth throughout tumor development (35). Nevertheless, the precise mechanisms by which gut bacteria contribute to the intratumoral microbiota remain not completely elucidated and warrant emphasis (3).

The TME is subject to regulatory influences from both intratumoral and gut microbiota, involving modulation of immune responses and modification of cancer cell metabolism (16). Modulation of the TME is achievable through gut microbiota-mediated regulation of intestinal epithelial barrier components, resulting in the activation of lymphoid organs. The gut microbiota may mediate its impact on the TME via metabolites or the immune system, thereby potentially altering the activities of the microbiota within the tumor (24, 36).

Comparable to the gut microbiota, the intratumoral microbiota exhibits the potential to modulate host immune responses. The gut microbiota intricately shapes the effectiveness of immune checkpoint blockade and the ensuing immune responses against tumors (37). Diverse interactions among intratumoral microbiota can trigger unique immune responses, suggesting a potential interplay with gut microbiota (15). Further investigation is warranted to clarify the interplay between intratumoral and gut microbiota.

## 3 Mechanistic insights into tumorigenesis and intratumoral microbiota

**Figure 2:**
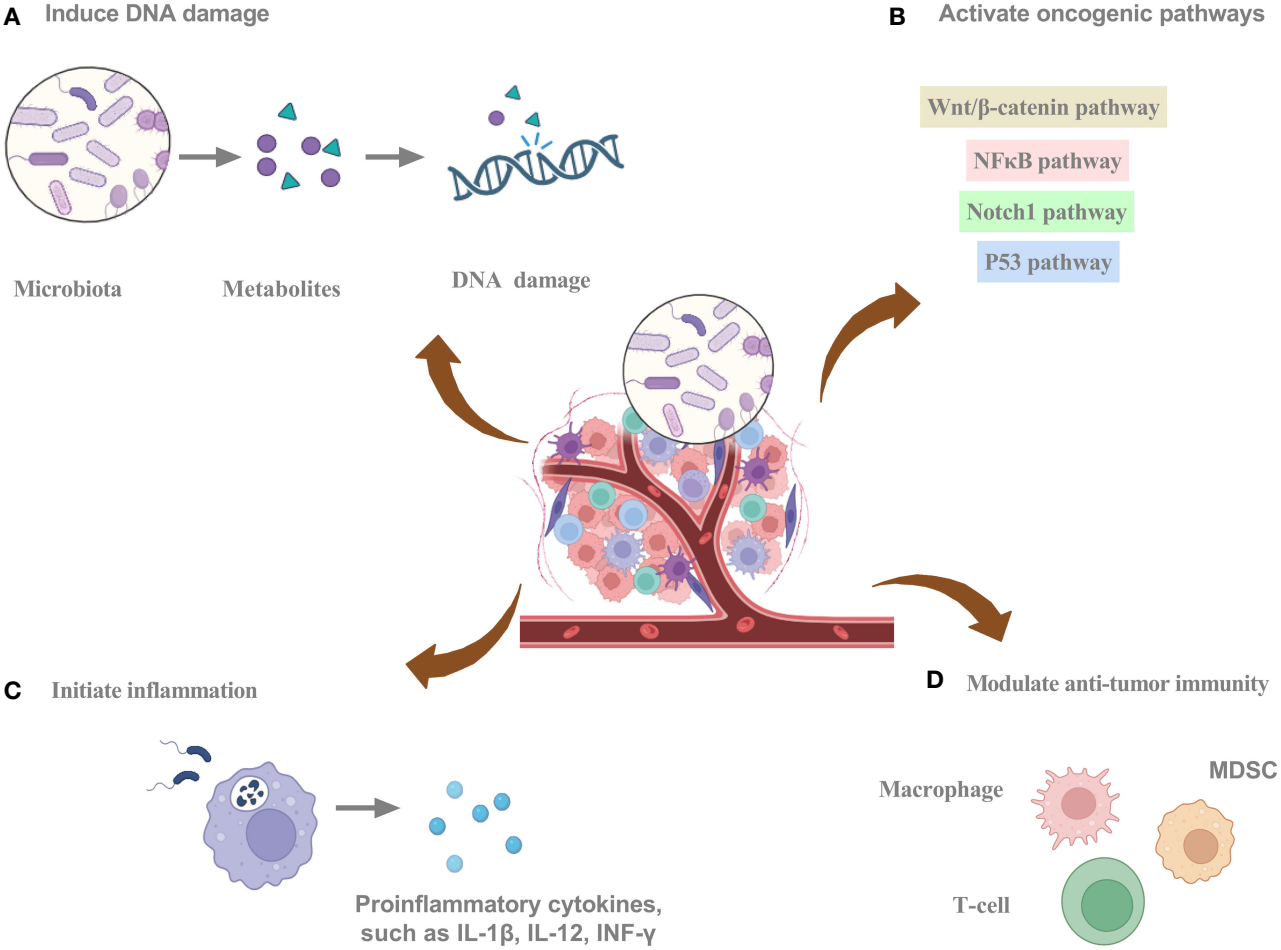
The potential mechanisms of intratumoral microbiota promoting tumorigenesis. **(A)** Intratumoral microbiota can secrete metabolites to induce DNA damage. **(B)** Intratumoral microbiota can activate oncogenic pathways. **(C)** Intratumoral microbiota can initiate inflammation. **(D)** Intratumoral microbiota can modulate anti-tumor immunity. Graphics created with BioRender.com.

Intratumoral bacteria can regulate cancer cell-intrinsic properties, such as mechanical stress, stem cell flexibility, epithelial-mesenchymal transition (EMT), and adhesion to endothelial cells, which can detrimentally impact the behavior of tumor cells in circulation. Intratumoral bacteria can regulate the extrinsic cancer milieu by releasing exosomes, thereby fostering metastasis, facilitating the breach of the vascular barriers for remote organ colonization, and contributing to the creation of a specialized premetastatic niche. Furthermore, they orchestrate the modulation of the adaptive and innate immune systems, ultimately dictating the resultant immune reaction (38). The intricate interplay between the intratumoral microbiota and cancer manifests in a multifaceted manner, exerting varied influences on cancer progression (**Figure 2**). These include promoting cancer growth and spread through increased mutagenesis, epigenetic modifications, modulation of oncogenes or oncogenic pathways, inflammation initiation, and immune response alteration (31, 38–40).

### 3.1 Induce DNA damage

Several bacterial species have evolved mechanisms to inflict DNA damage, which may instigate mutational events and ultimately promote carcinogenesis (41). Carcinogenic bacteria damage host DNA through a variety of mechanisms involving molecules, proteins, and metabolites. Fragile Bacteroidin exhibits the potential to cause DNA damage, thereby stimulating mutational events (3, 42). Single-cell RNA sequencing enables the identification of bacteria-associated host cells, their interactions, and the dysregulation of transcriptional pathways related to DNA damage repair, cell cycle, and the p53 signaling pathway (9). The production of colibactin by polyketide synthetase (pks)+ *Escherichia coli* can lead to DNA alkylation, provoking DNA damage and facilitating colorectal cancer progression (43). The pathogenic bacteria that adhere to the intestinal epithelium can induce episodes of diarrhea. The type 3 secretion system (T3SS) of these bacterial pathogens plays a crucial role in their interactions with intestinal epithelial cells, through which they can deliver genotoxin-UshA that damages the DNA of the host cells, contributing to the development of carcinogenesis (44). The involvement of microbes in instigating DNA damage through mutational processes is apparent. The mechanisms currently under consideration include *Escherichia coli*-mediated colibactin crosslinking, generating genotoxicity, and *Helicobacter pylori*-mediated aberrant cytidine expression. The exploration of mutational signatures through bioinformatics has opened the door to comprehending the processes underlying genomic alterations that drive oncogenesis. Microbes can elicit DNA damage that impacts the structure of the cancer genome, resulting in alterations to mutational spectra and mutational signatures (42). Additionally, the microbiota can convert numerous dietary metabolites into agents that damage DNA, and under conditions of dysbiosis, certain bacteria can produce toxins that cause DNA damage (3, 9, 45).

### 3.2 Activate carcinogenic pathways

Intratumor microbiota and their metabolites can influence signaling pathways that contribute to oncogenesis. *Fusobacterium nucleatum* has been implicated in the modulation of pathways and their associated molecules, exerting an influence on the landscape of pancreatic tumor development (46, 47). Through a Fap2-dependent pathway, *Fusobacterium nucleatum* engages with pancreatic cancer cells, inducing cytokine production. Through autocrine and paracrine pathways, cytokines stimulate cancer cell proliferation and enhance migration, ultimately propelling the evolution of the malignancy (47). Infections by bacteria lead to a substantial augmentation of signaling pathways, notably TNF, inflammatory responses, and hypoxia pathways. Furthermore, this fosters cancer cell progression through EMT and activation of the p53 pathway (9). Microbial metabolites can modulate signaling pathways such as transcription factor nuclear factor κB (NFκB) and Wnt/β‐catenin in tumor cells, thereby affecting tumor progression (3). In colorectal cancer, *Fusobacterium nucleatum* is recognized for its ability to trigger the initiation of the E-cadherin/β-catenin signaling cascades via FadA. This initiation eventuates in DNA damage, stimulation of cell growth, and augmentation of chk2 expression (48). CagA, a protein synthesized by *Helicobacter pylori*, can enter the host cell cytoplasm, triggering β-catenin signaling cascades, ultimately promoting the onset of gastric cancer (49). The involvement of Enterotoxigenic *Bacteroides fragilis* in breast cancer initiation is evident through both intraductal and intestinal colonization, emphasizing local and distant impacts. Elicitation of oncogenic effects by the *Bacteroides fragilis* toxin is potentially linked to the stimulation of the β-catenin and Notch1 signaling cascades (50).

### 3.3 Initiate inflammation

Chronic inflammation can elevate the likelihood of developing particular forms of cancer by activating inflammatory mediators and signaling cascades that promote tumor cell survival, proliferation, and invasion. Inflammatory mediators like ROS, cytokines, chemokines, and nitrogen species can facilitate tumor progression by fostering angiogenesis, elevating growth factor synthesis, and provoking the proliferation of cancerous cells (51, 52). Intratumoral bacteria can aggravate the inflammatory response, leading to the exacerbation of the disease (53). Intratumoral bacteria interacting with pattern recognition receptors (PRRs) can activate inflammatory pathways. Intratumoral bacteria may activate PRRs, leading to the secretion of cytokines and chemokines, the facilitation of angiogenesis, and immune cell recruitment (54, 55). An increased presence of *Fusobacterium* within tissues of head and neck squamous cell cancer has been linked to heightened inflammation and a less favorable prognosis. Moreover, complex interactions between competitive endogenous RNA networks and chromatin accessibility promote the development of microbiome-related inflammatory TME (56). *Fusobacterium nucleatum* can initiate the toll-like receptor 4 (TLR4)-mediated signaling cascade, which activates downstream signaling pathways and NFκB, leading to the induction of genes related to inflammation and the immune response (57). An elevated prevalence of *Enterobacteriaceae* is linked to heightened inflammatory activity, possibly attributed to their metabolizing inflammatory byproducts as an energy source (58). The secretion of virulence factors by *Escherichia coli* exacerbates the inflammatory response (59). The interplay between chronic inflammation and intratumoral bacteria requires further investigation.

### 3.4 Modulate anti-tumor immunity

Intratumoral microbiota can impact TME through several mechanisms, thus playing a role in tumorigenesis and cancer treatment (**Table 1**). Bacterial-induced modifications within the TME play a pivotal role in immunotherapy (69). Microbes within the TME elicit recognition by immune and cancer cells by presenting microbial antigens on their cell surfaces, stimulating an immune response and activating immune cells against the tumor (70). Moreover, some microbial antigens display structural resemblance to tumor antigens, activating immune cells that recognize these shared antigens. Consequently, the immune response triggered against microbial antigens can also target tumor cells expressing analogous antigens (71). In addition, some microbes in the TME can trigger immunogenic cell death, characterized by danger signal release and immune system activation, resulting in proinflammatory molecule secretion and tumor antigen presentation, facilitating an immune response against tumor cells (72). Furthermore, microbial component-mediated activation of PRRs boosts the immune response against tumors, eliciting the liberation of proinflammatory cytokines and heightened stimulation of immune cell activity (73, 74). Moreover, microbial-derived metabolites in the TME exert immunomodulatory effects by impacting immune cell behavior and remodeling the TME (75). Additionally, certain microbes in the TME can activate inhibitory checkpoints, diminish immune cell activity, and attenuate the anti-tumor immune response (72). Stimulated by intratumoral microbiota, the initiation of interleukin-17 production is triggered, fostering the infiltration of B cells into the complex microenvironment of tumor tissues. This intricately coordinated response emerges as a substantial factor in contributing to the progression of colon cancer. Within the milieu of colon cancer, polymorphonuclear neutrophils, recognized as highly abundant immune cells, have the potential to ameliorate microbial dysbiosis in colon cancer tissues. This is manifested by a decrease in tumor-associated *Akkermansia* and a concurrent increase in the prevalence of *Proteobacteria* (76). Within microsatellite instability-high colorectal cancers, the Fusobacterium nucleatum-enriched subset exhibits heightened tumor invasion. Furthermore, specific features within the immune microenvironment become evident, highlighting a significant reduction in FoxP3+ T cells spanning the entire tumor and a notable increase in the proportion of M2-polarized macrophages positioned within the tumor (77).

**Table 1:** Functional roles of intratumoral microbiota in the modulation of the tumor microenvironment.

Intratumoural microbiota	Mechanism	Cancer	References
*Bifidobacterium*	The localized delivery of *Bifidobacteria* efficiently triggers STING signaling and enhances the initiation of crossover events in dendritic cells after anti-CD47 treatment	Digestive tract cancer	(60)
*Enterococcus faecalis*	The pancreatic ductal adenocarcinoma microbiome orchestrates TAM programming through TLR signaling, inducing immune tolerance	Pancreatic cancer	(61)
*Fusobacterium* and *Treponema*	*Fusobacterium* and *Treponema* species were notably associated with macrophages and aneuploid epithelial cells, resulting in the upregulation of JAK-STAT signaling, interferon, and inflammatory response pathways	Oral squamous cell carcinoma	(62)
*Saccharopolyspora*, *Pseudoxanthomonas*, and *Streptomyces*	The tumor microbiome’s diversity and the inclusion of *Saccharopolyspora*, *Pseudoxanthomonas*, and *Streptomyces* species within tumors could potentially enhance the anti-tumor immune response by aiding in the recruitment and activation of CD8+ T cells	Pancreatic cancer	(63)
*Streptococcus*	Tissue densities show a positive correlation of GrzB+ and CD8+ T cells with *Streptococcus* and a negative correlation of FOXP3+ and CD4+ T cells with *Streptococcus*	Esophageal squamous cell carcinoma	(64)
*Dialister* and *Casatella*	*Dialister* and *Casatella* displayed robust associations with MSI. *Dialister* exhibited positive correlations with CD3E and CD8E, indicating overall tumor-infiltrating lymphocytes and cytotoxic T cells	Colorectal cancer	(65)
*Fusobacterium nucleatum*	Fusobacterium nucleatum is inversely associated with CD3, signifying immunosuppression	Colorectal cancer	(65)
*Lactobacillus*	*Lactobacillus* prevalence within the tumor may impact local microbiome diversity, leading to elevated PD-L1 expression in ECs and TAMs	Esophageal squamous cell carcinoma	(66)
*Lachnospiraceae*	*Lachnospiraceae* bacteria within tumors enzymatically degrade lyso-glycerophospholipids, sustaining CD8+ T cell immune surveillance and defending against colorectal carcinogenesis	Colorectal cancer	(67)
*Acinetobacter baumannii*	*Acinetobacter baumannii* is prominently enriched in the immune-enriched subtype, marked by elevated stromal and immune scores, and a higher presence of CD81 T cells and M1-type macrophages, fostering a proinflammatory microenvironment	Ovarian cancer	(68)
*Fusobacterium nucleatum*	*Fusobacterium nucleatum*, enriched in immune-deficient patients, drives tumorigenesis through FadA adhesin and outer membrane vesicle, offering tumor protection by binding to inhibitory receptors	Ovarian cancer	(68)

MSI-H, High-level microsatellite instability; TAM, tumor-associated macrophage; TLR, Toll-like receptor.

The microbiota may exert a significant impact on an immunosuppressive TME in pancreatic ductal adenocarcinoma (78). By translocating to the pancreas, the gut microbiome can initiate the formation of a TME exhibiting immunosuppressive, promoting tumorigenesis and metastatic spread, consequently impairing the potency of modulators targeting immune checkpoints (78). The increase of immune cells with immunosuppressive properties, such as myeloid-derived suppressor cells (MDSCs), regulatory T cells (Tregs), along with cytokines, obstruct TILs from penetrating the tumor site (78, 79). In oral cavity tumors, *Fusobacterium nucleatum* load exhibited a negative correlation with immune markers. Elevated *Fusobacterium nucleatum* levels were associated with decreased B lymphocytes, T helper lymphocytes, M2 macrophages, and fibroblasts. In tumors exhibiting a high load of *Fusobacterium nucleatum*, significant reductions were noted in the expressions of Toll-like receptor (TLR) 4 and OX40 ligand (TNFSF4). Significantly, TNFSF9 receptor (TNFRSF9) expression underwent a marked decrease, mirroring an escalation in its ligand (TNFSF9) expression with the mounting *Fusobacterium nucleatum* load. Simultaneously, there was a marked elevation in the levels of the pro-inflammatory cytokine IL-1ß (17). The presence of intratumoral microbiota has been identified as a pivotal factor in fostering an immunosuppressive TME by selectively recruiting specific immunosuppressive cellular populations, including Tregs, MDSCs, and TAMs. Consequently, this orchestrated recruitment acts as a deterrent to the efficacious infiltration of TILs (3, 17, 80). The depletion of CD4+ T cells of the Th1 subtype and CD8+ T cells with cytotoxic activity, accompanied by a shift towards Th2 T cells, as well as the shift of tumor-associated macrophages (TAMs) towards the M2 phenotype associated with immunosuppression, are associated with immune suppression and an unfavorable TME (78, 81, 82). The fibrogenic reprogramming of pancreatic ductal adenocarcinoma stellate cells results in a dense fibrotic stroma, impeding the penetration of therapeutic drugs and immune cells into the tumor locale. Furthermore, the activated pancreatic stellate cells recruit immunosuppressive cells, establishing a TME exhibiting immunosuppressive features, thus facilitating tumor growth and dampening effective immune reactions targeting tumors (78, 83).

Some microorganisms can interface with immune cells in the TME, potentially modulating their activity (11, 24). *Fusobacterium nucleatum* can impede the cytotoxicity exhibited by natural killer (NK) cells against tumors. *Fusobacterium nuclei* strains inhibit the cytotoxicity of NK cells by engaging with the Fap2 protein, leading to subsequent attachment to the inhibitory receptor TIGIT. Tumors exploit the Fap2 protein derived from *Fusobacterium nucleatum* to promote immune escape via TIGIT-mediated inhibition of immune cell function (84). *Fusobacterium nucleatum* can interact with carcinoembryonic antigen-related cell-adhesion molecule 1 (CEACAM1), thereby exerting an inhibitory effect on the function of T and NK cells (85). Commensal microbiota-mediated modulation of γδ T cell functionality impacts immune reactivity. Specifically, the microbiota elicits the activation of T cells, particularly those with the Vγ6+Vδ1+ phenotype, in lung cancer. These γδ T cells facilitate neutrophil penetration and stimulate the growth of tumor cells, thereby influencing the TME and tumor progression (86). Within colorectal carcinoma tissue, an inverse correlation has been observed between the prevalence of *Fusobacterium nucleatum* and the abundance of CD3+ T-cell count. A reduced CD3+ T-cell density can facilitate tumor progression by decreasing immune surveillance and impairing anti-tumor activity (87).

## 4 The potential of intratumoral microbiota for tumor therapy

**Figure 3:**
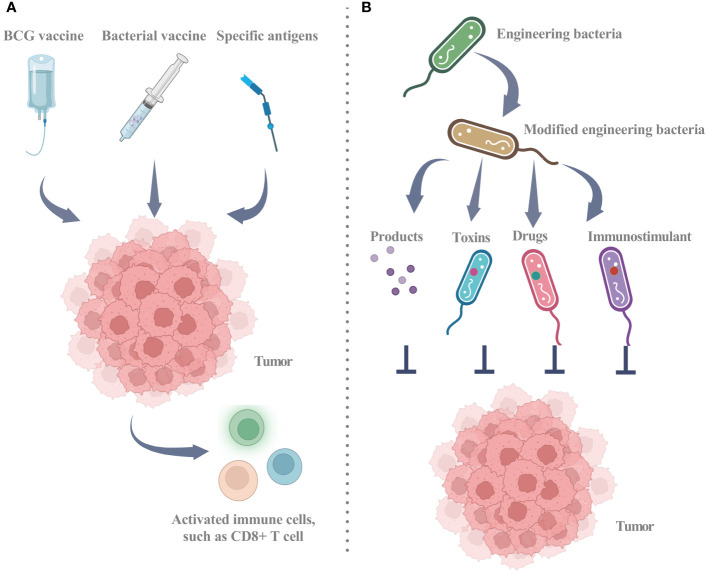
Utilizing intratumor microbiota for clinical treatment strategies. **(A)** Utilizing biological agents, such as the BCG vaccine and multiple bacterial vaccines, involves the use of either dead or living bacteria to recruit active immune cells, including CD8+ T cells, thereby triggering an anti-tumor immune response. The strategic utilization of specific antigens stands out as a pivotal mechanism to activate the immune system, fostering a heightened and robust CD8+ T cell response against cancer cells. **(B)** Engineered bacteria as a tool for tumor inhibition through the release of targeted products or reactions and as vehicles for delivering toxins, immunostimulants, or other drugs. Engineered bacteria can be programmatically designed to release targeted products or undergo specific reactions near tumor cells, encompassing toxins for direct cancer cell eradication, anti-angiogenic factors to impede vascular growth within tumors, or other agents impeding tumor progression. Engineered bacteria emerge as promising vehicles for the delivery of therapeutic agents, encompassing toxins, immunostimulants for immune response amplification against cancer, and conventional drugs. This targeted delivery system is designed to heighten treatment specificity and efficacy while mitigating potential harm to healthy tissues. BCG: Bacillus Calmette-Guérin. Graphics created with BioRender.com.

Current research has established the considerable contribution of the microbiome to diverse aspects of cancer, such as oncogenesis, therapeutic response, and drug resistance (41). Strategic alteration of the gut microbiota holds promise for mitigation and management of cancer. However, the therapeutic potential of intratumoral microbiota warrants further investigation (22). Intratumoral microbiota may exert adverse or favorable effects on cancer therapy, depending on the underlying therapeutic mechanism (**Figure 3**; **Table 2**) (93). Two principal approaches for microbial-based treatments have progressed to the clinical stage. The first approach employs living or inactivated bacteria to stimulate an immune response via targeting specific antigens. The Bacillus Calmette-Guérin (BCG) vaccine, various bacterial vaccines, and the implementation of live, attenuated, double-deleted Listeria monocytogenes are notable examples of this strategy. The second strategy involves utilizing bacteria as carriers capable of the controlled release of immunostimulants, toxins, and other pharmaceutical agents. Engineered bacteria can elicit an anti-tumor response or serve as carriers for therapeutic applications. Through genetic modifications, engineered bacteria can release products or facilitate specific reactions that impede the progression of tumors. Furthermore, engineered bacteria can function as carriers for the targeted delivery of toxins, immunostimulants, or other therapeutic substances (11).

**Table 2:** Exploring therapeutic implications of intratumor microbiota.

Intratumoural microbiota	Therapy	Cancer	References
*Dialister* and *Prevotella*	Colorectal tumors with MSI-H show higher levels of *Dialister* and *Prevotella*, correlating with increased mutation burden and improved response to anti-PD-1 therapy	Colorectal cancer	(65)
*Streptococcus*	Increased *Streptococcus* in TME links to an activated tumor immune microenvironment, potentially boosting neoadjuvant chemotherapy with immune checkpoint inhibitor efficacy	Esophageal squamous cell carcinoma	(64)
*Bifidobacterium*	Accumulation in the tumor microenvironment empowers *Bifidobacterium* to boost local anti-CD47 immunotherapy	Digestive tract cancer	(60)
*Fusobacterium nucleatum*	The chemotherapeutic 5-fluorouracil serves as a potent inhibitor of *Fusobacterium nucleatum* colorectal cancer isolates	Colorectal cancer	(88)
*Acinetobacter jungii*	The positive correlation observed between *Acinetobacter jungii* presence and PD-L1 expression	Non-small cell lung cancer	(89)
*Haemophilus parainfluenzae*	In stage IV patients, the response to targeted therapy or chemotherapy showed a negative correlation with the presence of *Haemophilus parainfluenzae*	Non-small cell lung cancer	(89)
*Collinsella*, *Alistipes*, *Christensenella*, *Faecalibacterium*, *Ruminococcus*, *Pavimonas*, and *Akkermansia*	*Collinsella*, *Alistipes*, *Christensenella*, *Faecalibacterium*, *Ruminococcus*, *Pavimonas*, and *Akkermansia* showed significant associations with responses to neoadjuvant chemoradiotherapy	Rectal cancer	(90)
*Pseudomonas*, *Serratia*, and *Streptococcus*	Patients showcasing elevated mitotane levels were notably associated with adrenocortical carcinoma featuring a substantial prevalence of *Pseudomonas* and *Serratia*, or a diminished presence of *Streptococcus*	Adrenocortical cancer	(91)
Gammaproteobacteria	Gemcitabine resistance is linked to intratumoral *Gammaproteobacteria* expressing the bacterial enzyme cytidine deaminase	Pancreatic ductal adenocarcinoma	(92)

MSI-H, High-level microsatellite instability.

Intratumoral bacteria have been implicated in altering tumor cell responsiveness to chemotherapy. Specific bacterial enzymes have been noted to mediate the metabolic conversion of gemcitabine into an inactive metabolite. The colonization of pancreatic tumors by *Gammaproteobacteria* has been correlated with their ability to degrade gemcitabine, which subsequently contributes to an enhanced chemoresistance of the tumor (94). In colon cancer, intratumoral *Gammaproteobacteria* facilitated resistance to gemcitabine through the synthesis of bacterial cytidine deaminase (CDDL) enzyme and was subsequently eradicated through the concurrent administration of ciprofloxacin (92). Analysis of taxonomic distributions revealed higher levels of *Gammaproteobacteria* in cholangiocarcinoma tumor tissues resistant to low-dose gemcitabine, low-dose cisplatin, and high-dose gemcitabine, while the abundance of Actinobacteria was lower in low-dose gemcitabine and high-dose gemcitabine resistant groups (95). The intratumoral presence of CDDL-expressing bacteria facilitates the metabolism of gemcitabine into 2’2-difluorodeoxyuridine (dFdU), thus preventing the inhibition of DNA replication within malignant cells. The reduction in bacterial-mediated resistance upon depletion of NupC, the transporter for bacterial nucleosides, in bacteria with active CDDL expression, indicates the involvement of NupC in the internalization of gemcitabine by the bacteria (96). Post neoadjuvant chemotherapy, a substantial augmentation of *Pseudomonas* within breast tumors was witnessed. Moreover, breast malignancies in individuals experiencing distant metastatic spread demonstrated an elevated prevalence of *Staphylococcus* and *Brevundimonas* (97). Variations in intratumoral microbiota signatures distinguish responders from non-responders to neoadjuvant chemoimmunotherapy (NACI) in patients with esophageal squamous cell carcinoma. Responders displayed heightened levels of tumor-resident *Streptococcus*, establishing a positive correlation with the increased infiltration of CD8+ T cells and GrzB+ T cells. Fecal microbial transplantation (FMT) from NACI responders restructured the intratumoral microbiota composition, resulting in *Streptococcus* enrichment in tumor tissues, increased infiltration of CD8+ T cells, and the promotion of positive results with anti-PD-1 therapy (64).

Intratumoral microbiota may exert both immunostimulatory and immunosuppressive effects on anti-tumor immunity, with the potential to promote the advancement of cancer by inducing processes such as heightened production of ROS, fostering an anti-inflammatory milieu, impairing T cell function, and instigating immunosuppressive responses (3). To elucidate the correlation between a specific intratumor microbial signature and the response to immunotherapy, a comparative analysis of metastatic melanomas was carried out. Examination of distinct microbial taxa profiles in patients, including immune checkpoint inhibitor responders (n=29) and non-responders (n=48), unveiled noteworthy distinctions. There were 18 high-abundance taxa and 28 low-abundance taxa among responders compared with non-responders. Notably, responders showed an increased abundance of *Clostridium*, whereas non-responders exhibited a higher *Gardnerella vaginalis* (14). The attenuated vaccine BCG, originating from *Mycobacterium bovis*, has been implemented in clinical therapies for bladder cancer (98). The efficacy of traditional cancer treatments, including radiation and chemotherapy, is diminished in areas with low oxygen levels. *Clostridium novyi-NT* can thrive in this oxygen-deprived environment, facilitating the destruction of hypoxic and necrotic regions within tumors. *Clostridium novyi-NT* bacteria can replicate and selectively target cancer cells. The production of toxins by these bacteria can inflict damage upon tumor cells and incite an immune response leading to the eradication of the tumor (99). In the phase I trial (NCT01924689) involving 24 individuals with solid neoplasms, the intratumoral administration of *Clostridium novyi-NT* initiated the activation of bacterial spores, leading to a 42% incidence of tumor mass breakdown. Among the evaluated cohort of 22 individuals, 41% exhibited a decline in injected tumor dimensions, and 86% showed a stable disease (100). *Bifidobacterium* fosters the effectiveness of anti-CD47 immunotherapy through its accumulation within the TME, mediated by interferon-dependent mechanisms and the activation of the Stimulator of interferon genes (STING) pathway (60). Following bacterial ablation, the pancreatic ductal adenocarcinoma TME underwent immunogenic reprogramming, characterized by diminished MDSCs and heightened M1 macrophage differentiation, facilitating the Th1 polarization in CD4+ T cells and stimulating the induction of CD8+ T-cell. Augmented PD-1 levels following bacterial ablation were associated with improved efficacy of immunotherapy. An abundant and distinct microbiome triggers the differentiation of suppressive monocytic cells in pancreatic cancer by selectively activating Toll-like receptors (TLRs), ultimately resulting in T-cell anergy (61).

Disruptions in the microbiota contribute to the accumulation of toxic metabolites and the persistence of inflammatory reactions, thus fostering cancer development and the evolution of treatment resistance (2). Remodeling intratumoral microbiota has emerged as a promising avenue for potential therapeutic strategies. Probiotics, antibiotics, and fecal microbiota transplantation are the prevailing techniques utilized for systemic microbiota, offering a feasible avenue for their application in targeting the intratumoral microbiota associated with cancer (31, 101).

## 5 Conclusions

**Figure 4:**
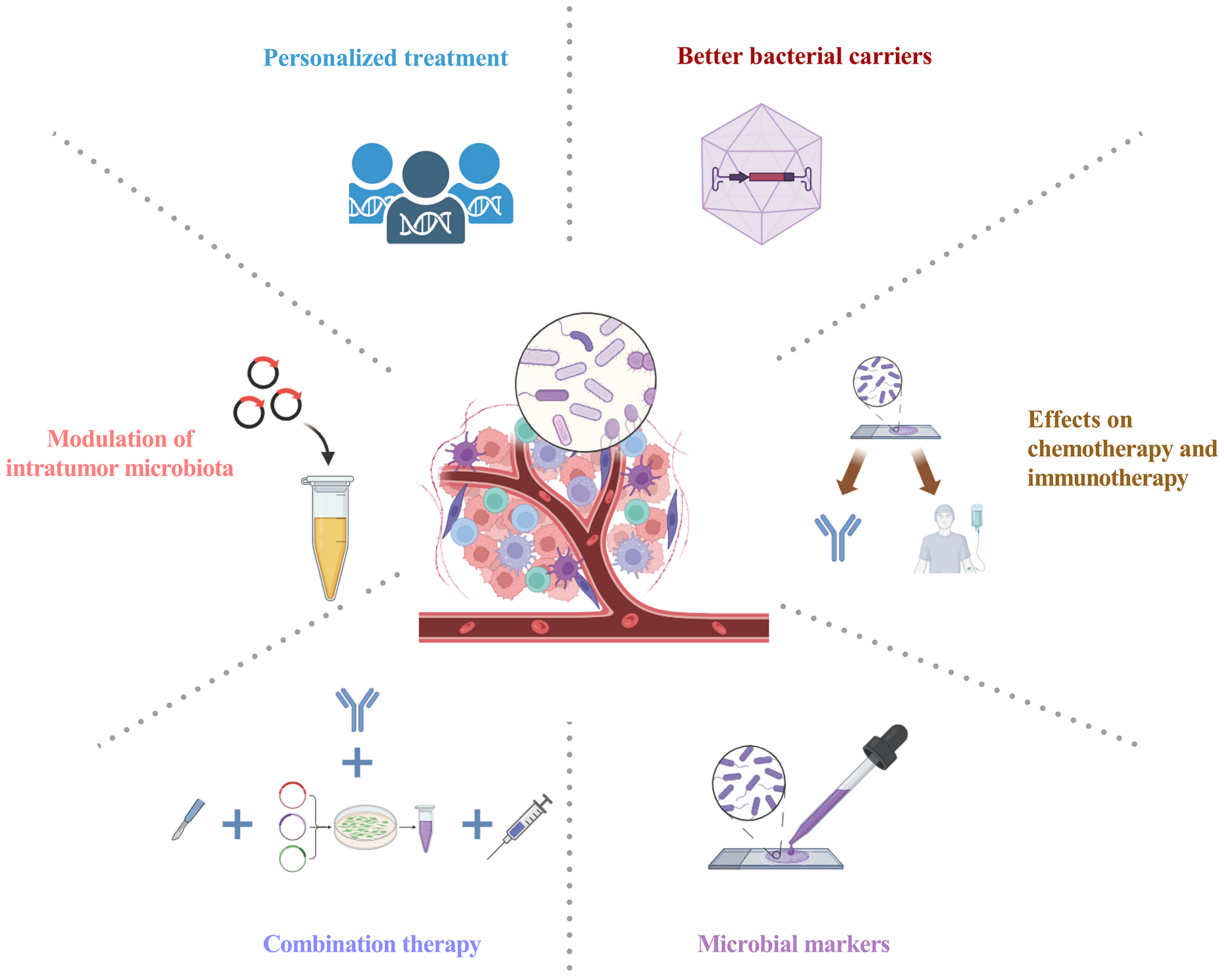
Intratumoral microbiota: prospects for clinical application. Personalized treatment: Integrating advanced sequencing techniques allows for the comprehensive analysis of intratumoral microbiota, shedding light on microbial-derived antigens and paving the way for personalized treatment modalities; Modulation of intratumor microbiota: Leveraging probiotics, antibiotics, and targeted interventions stands as a promising strategy for intratumoral microbiota modulation, aiming to reinstate a harmonized microbial community; Combination therapy: Combining antibiotics or bacterial therapies with other anti-tumor treatments, such as chemotherapy or immunotherapy, seeks to optimize cancer therapy by targeting both tumor cells and the intratumor microbiota; Better bacterial carriers: This innovative strategy maximizes specific bacterial attributes, utilizing advanced carriers for precise tumor therapeutics with strong targeting, lower infection risk, and superior payload efficiency; Microbial markers: Utilizing intratumoral microbiota for early cancer diagnosis, prognosis, and monitoring; Effects on chemotherapy and immunotherapy: Impact of intratumoral microbiota on chemotherapy and immunotherapy, evaluating efficacy, tolerability, and toxicity. Graphics created with BioRender.com.

Amidst the burgeoning interest in unraveling the relationship between gut microbiota and tumors, attention is now directed toward probing the effects of intratumoral microbiota on tumorigenesis and its implications for cancer treatment. Advances in techniques for analyzing the gut and tumor microbiome have enhanced the understanding of the microbiome’s impact on human health. Nevertheless, the exploration of intratumoral bacteria is still in its preliminary phase. Recent findings demonstrate the widespread occurrence of intratumor microbiota in various tumor types. The complexity and ambiguity of the host-intratumoral microbiota interplay necessitate future studies to improve the understanding of the intratumor microbiota in carcinogenesis. Intratumoral microbiota exerts immunomodulatory effects within the TME, influencing tumor outcomes by promoting inflammatory responses or regulating anti-tumor activity. Intratumoral microbiota exerts a significant influence on therapeutic effectiveness, offering novel avenues for cancer therapy, diagnostic and prognostic assessment, and potential therapeutic targets (**Figure 4**). In particular, the complex interactions among intratumoral microbiota, antitumor immunity, and therapeutic efficacy in tumors require further investigation. Comprehensive profiling of distinct intratumor microbiota holds promise for manipulating these bacterial communities to advance cancer treatment. Further research into the molecular mechanisms of intratumoral microbiota is also necessary. Targeting the intratumoral microbiota presents opportunities for potential universal therapies and synergistic combination approaches with approved chemotherapeutics and immunotherapies. Considering the significant impact of microbial metabolites, integrating microbiome and metabolome profiles may emerge as a pivotal approach for personalized therapies. Undoubtedly, the significance of intratumoral microbiota within tumor biology is poised to assume a pivotal role in forthcoming decades of carcinogenesis investigations.

## Author contributions

JW: Conceptualization, Data curation, Formal analysis, Investigation, Methodology, Project administration, Resources, Visualization, Writing – original draft, Writing – review & editing. PZ: Conceptualization, Data curation, Formal analysis, Methodology, Project administration, Resources, Software, Writing – original draft. WM: Conceptualization, Supervision, Validation, Writing – review & editing. CZ: Funding acquisition, Supervision, Writing – review & editing, Writing – original draft.
